# Whole food or processed food or mutated recombinant protein?

**DOI:** 10.1186/2045-7022-1-S1-S77

**Published:** 2011-08-12

**Authors:** Anna Nowak-Wegrzyn

**Affiliations:** 1 Mount Sinai School of Medicine, New York, USA

## 

Diverse therapeutic strategies for food allergy are under investigation including food allergen-specific and non-specific approaches. Allergen-specific approaches include immunotherapy with native food allergens, immunotherapy with mutated recombinant allergens, and diets containing extensively heated milk or egg. Native allergen immunotherapy can be administered via oral, sublingual, subcutaneous, or epicutaneous route. Oral immunotherapy trials with native food allergens such as milk, peanut, or egg reported that approximately 50-75% of the treated subjects reached and tolerated the maintenance dose. Failure of desensitization occurred in about 10-20% of the treated subjects and might be associated with the most severe and likely permanent food allergy phenotype, as opposed to the successful desensitization and tolerance that might be associated with a transient clinical phenotype and higher chances of spontaneous resolution of food allergy. Food allergens can be altered to decrease their allergenicity and lower the risk of acute adverse reactions. Immunotherapy with mutated recombinant peanut proteins, which have decreased IgE-binding activity, co-administered within heat-killed E.coli to generate maximum immune response had a strong protective effect in the mouse model of peanut anaphylaxis. This vaccine is being currently evaluated in the phase I clinical trials in adults with peanut allergy. Heating and processing changes food protein conformation and affects how food proteins are digested and transported via the gut barrier. Extensively heated (baked) milk and egg are tolerated by approximately 70% of the milk or egg allergic children. Diets containing extensively heated (baked) milk and egg represent an alternative approach to food oral immunotherapy and are already changing the paradigm of strict dietary avoidance for food-allergic patients.

